# Guifu Dihuang Pills Ameliorated Mucus Hypersecretion by Suppressing Muc5ac Expression and Inactivating the ERK-SP1 Pathway in Lipopolysaccharide/Cigarette Smoke-Induced Mice

**DOI:** 10.1155/2021/9539218

**Published:** 2021-11-03

**Authors:** Huanhuan Zhang, Wenying Yu, Liting Ji, Yusen Zhong, Yiyou Lin, Huazhong Ying, Chenhuan Yu, Changyu Li

**Affiliations:** ^1^School of Pharmaceutical Sciences, Zhejiang Chinese Medical University, Hangzhou 310053, China; ^2^Zhejiang Provincial Laboratory of Experimental Animal's & Non Clinical Laboratory Studies, Hangzhou Medical College, Hangzhou 310013, China

## Abstract

Mucus hypersecretion is a hallmark of chronic obstructive pulmonary disease (COPD) and is associated with increasing sputum production and declining pulmonary function. Therefore, reducing mucus secretion can be a new therapeutic opportunity for preventing COPD. The Guifu Dihuang pill (GFDHP) is a classical Chinese medicine and has been used as an immunoregulator for treatment of kidney yang deficiency syndrome, including hypothyroidism, adrenocortical hypofunction, chronic bronchitis, and COPD, for more than 2000 years. However, the protective effects and mechanisms of GFDHP against mucus hypersecretion in COPD remain obscure. The aim of the present study was to explore the inhibitory effects of GFDHP on lipopolysaccharide/cigarette smoke- (LPS/CS-) induced Mucin5ac (Muc5ac) overproduction and airway goblet cell hyperplasia in mice. The mice were randomly assigned into 6 groups: control, model, GFDHP-L, GFDHP-M, GFDHP-H, and dexamethasone. The mice were given LPS twice through intranasal inhalation and then exposed to CS daily for 6 weeks. Three doses of GFDHP were orally administered daily during the last 3 weeks of the experiment. Pulmonary function was examined with an EMKA pulmonary system, and pulmonary hyperpermeability and lung damage were evaluated with an *in vivo* imaging system. Inflammatory cells and cytokines in bronchoalveolar lavage fluid (BALF) were detected with a cell count analyzer and though ELISA analysis, respectively. Lung pathological changes and airway goblet cell hyperplasia were analyzed with hematoxylin and eosin and Alcian blue periodic acid Schiff staining. The protein expression levels of Muc5ac and extracellular signal-regulated kinase (ERK)-specificity protein1 (SP1) signaling pathway were measured with Western blot and immunohistochemistry. The results demonstrated that GFDHP improved pulmonary function and suppressed mouse pulmonary hyperpermeability and edema. GFDHP suppressed inflammatory cell infiltration and cytokine release in BALF, thereby elevating pulmonary function. It ameliorated lung pathological changes and airway goblet cell hyperplasia, and suppressed expression levels of Muc5ac mRNA and protein and phospho-ERK and SP1 levels in the lung tissues of the COPD mice. In conclusion, GFDHP inhibited mucus hypersecretion induced by LPS/CS by suppressing the activation of the ERK-SP1 pathway.

## 1. Introduction

As a worldwide burden, chronic obstructive pulmonary disease (COPD) affects more than 255 million people and has been one of the major causes of mortality and morbidity in smokers [1]. In China, the prevalence rate of COPD was 8.6%, accounting for 99.9 million adults [2]. It is a chronic progressive disease characterized by airflow limitation and persistent respiratory symptoms mainly caused by instance cigarette smoking (CS) or significant exposure to noxious particles or gases, which result in airway and/or alveolar abnormalities [3]. Glucocorticoids are commonly used in clinics but have systemic side effects that cannot be ignored during long-term treatment. Owing to the complex pathology and pathogenesis of COPD, no effective strategies for its prevention and treatment have been established. Recent evidence showed that, apart from persistent airway inflammation and bronchial wall remodeling, goblet cells hyperplasia and mucus hypersecretion contribute to irreversible airflow limitation and pulmonary function deterioration [4]. Therefore, reducing airway inflammatory response and suppressing mucus hypersecretion may be an effective approach for COPD treatment.

The Guifu Dihuang pill (GFDHP, Chinese name 桂附地黄丸), composed of eight herbs, is a classical Chinese medicine for the treatment of kidney yang deficiency (KYD) syndrome [5]. It is also called the Jinkui Shenqi pill because it was first recorded in the Synopsis of Golden Chamber written by Zhang Zhongjing approximately 2000 years ago [6]. It had been used as an immunomodulator for preventing renal and immune hypofunction, particularly chronic nephritis, diabetes, hypothyroidism, adrenocortical hypofunction, sexual hypofunction, chronic bronchitis, asthma, and COPD [7]. According to the theory of Traditional Chinese Medicine (TCM), the long-term KYD can induce gradually deteriorating “lung inflation” and “phlegm retention,” which in turn accelerate the development of the KYD disease and eventually lead to the emergence of a malignant phenotype [5]. Thus, the eight herbs in GFDHP, most of which treat lung-distension and phlegm retention, would be helpful in recovering pulmonary ventilation function in patients with COPD [8]. Recently, GFDHP mainly contains various alkaloids and phenolic acids, such as aconitine, loganin, benzoylmesaconine, paeoniflorin, paeonol, and gallic acid [9–12]. The medicine not only improves the function of hypothalamic pituitary adrenal axis, but also inhibits allergen-induced airway inflammation and regulates T helper 1/2 imbalance [13]. It can improve pulmonary ventilation function and walking ability in patients with COPD [14]. After GFDHP was administered in combination with *β*2-adrenoceptor agonists or glucocorticoids, obvious synergistic effects were seen on lung function, clinical symptoms, and quality of life of patients with COPD [15]. The use of GFDHP in treating “phlegm-type cough and asthma” syndrome in China [13] indicates that it can significantly suppress mucus secretion in patients with asthma. However, whether GFDHP inhibits phlegm formation and improves pulmonary function remains unknown. Therefore, in our study, the effects and underling mechanisms of GFDHP on mucus hypersecretion and airway inflammation were investigated in mice challenged with lipopolysaccharide (LPS) and cigarette smoke (CS).

## 2. Materials and Methods

### 2.1. Drugs and Reagents

Commercially available GFDHP (Lot No. 180201) was purchased from Zhongjing Wanxi Pharmaceutical Co., Ltd., (Henan, China). The raw materials and composition ratios of the eight herbs were as follows: cinnamon (Rougui, *Cinnamomum verum*, 20 g), processed aconite (Fuzi, *Aconitum carmichaeli Debeaux*, 20 g), prepared Rehmannia glutinosa (Dihuang, *Rehmannia glutinosa*, 160 g), processed Corni Fructus (Shanzhuyu, *Cornus officinalis*, 80 g), Cortex of the Peony Tree Rote (Mudanpi, *Paeonia suffruticosa*, 60 g), Dioscorea root (Shanyao, *Dioscorea oppositifolia*, 80 g), Poria (Fuling, *Scierotium Poreae Cocos*, 60 g), and Alismatis rhizoma (Zexie, *Alisma plantago-aquatica subsp*. Orientale, 60 g). Suspensions were prepared by dissolving the pills in purified water and stored in a biosafety cabinet (to make it endotoxin-free) before the animal experiment. The quality of GFDHP was controlled through UPLC-Q/TOF-MS fingerprint analysis. The results are shown in the Supplementary Materials. The hongmei cigarettes (10 mg of tar and 0.8 mg of nicotine per cigarette) were obtained from Hongta Tobacco (Group) Co., Ltd., (China). LPSs (Lot. 039M4004V) were obtained from Sigma. Dexamethasone acetate (H12020686) was obtained from Tianjin Tianyao Pharmaceuticals (China). Other analytical reagents used in this study were purchased from Shanghai Aladdin Company or Hangzhou Chemical Reagent Co., Ltd., (China).

### 2.2. Animals and Treatments

Eight-week-old SPF male C57BL/6 mice (22–24 g) were supplied by Zhejiang Laboratory Animal Center, China. The mice were housed in a controlled room with constant temperature (21 ± 2°C), light (12 h light/12 h dark), and humidity (50% ± 10%). All experiments were performed in accordance with the protocols (No. 2018R0327) approved by Zhejiang department of the animal care committee.

**Figure 1:**
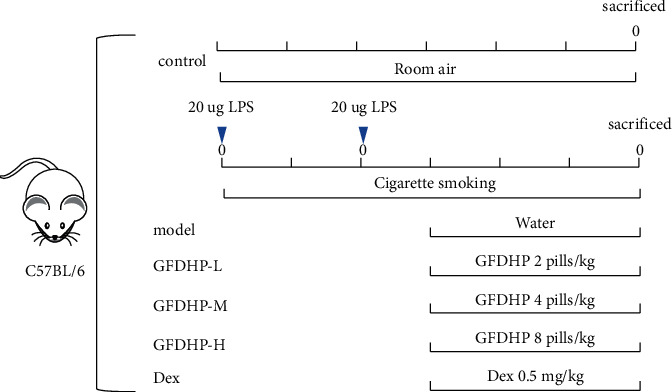
Animal experimental protocol.

The mice were acclimatized for 7 days and randomly assigned into six groups (15 mice per group): normal control, model, low-dose GFDHP-treated (GFDHP-L), middle-dose GFDHP-treated (GFDHP-M), high-dose GFDHP-treated (GFDHP-H), and dexamethasone-treated (Dex). The COPD model was prepared according to a previously reported method [11]. Approximately 50 *μ*l of saline containing 20 *μ*g of LPS was administered to the mice through intranasal inhalation at the 1st day and the 15th day of the experiment, except the mice in the normal control group. The mice challenged with LPS were exposed to CS (six cigarettes/30 min, 1 h/session, twice/day, and 6 days/week) in a Plexiglas chamber (0.79 m × 0.40 m × 0.30 m) for 6 weeks except in days when LPS was administered. GFDHP (0.4, 0.8, and 1.6 g/kg, respectively) and Dex (0.5 mg/kg) were administered orally 1 h before CS exposure during the last 3 weeks of the experiment. The timeline is shown in Figure 1. The low dose of GFDHP was equal to half clinical dosage after conversion and was selected according to our previous study [16]. Approximately 24 h after the final administration, the pulmonary function of mice was examined and then all animals were sacrificed with moderate anesthesia (isoflurane). The bronchoalveolar lavage fluid (BALF) and lung tissues were collected for further investigation.

### 2.3. Pulmonary Function Examination

The mice were placed in the plethysmograph of an EMKA pulmonary system (EMKA, France) directly for the measurement of peak expiratory flow (PEF), breathing frequency (*f*), enhanced pause (PenH), and other parameters according to the manufacturer's protocol. Values measured during the 5 min sequence were averaged.

### 2.4. *In Vivo* Imaging for Lung Damage Analysis

Five mice in each group were injected with 100 *μ*l of 1% Evans Blue dye (in normal saline) through the tail vein and then anesthetized with 2.5% isoflurane. The fluorescence images of the mice were acquired with an *in vivo* imaging system (OPT plus, Japan) with a 658 nm excitation filter, 719 nm emission filter, and 1 s acquisition time [17]. Fluorescence intensities were analyzed with OPT inherent software and used in investigating the degree of mouse lung injury.

### 2.5. Cell Count and Cytokine Detection in BALF

The left lung was camped, and the right lung was washed with 0.5 ml of sterile phosphate buffered saline (PBS) three times to obtain the lavage. Cell lysis was prevented by placing the recovered BALF samples on ice. Then the samples were centrifuged at 3000 rpm, 4°C for 10 min. The cell pellets were resuspended in 0.3 ml of PBS with 0.1% bovine serum albumin and the number of neutrophils, lymphocytes, and leukocytes was detected though automatic whole blood count (Tecom, China). The levels of cytokines IL-1*β*, IL-6, and TNF-*α* in the BALF were detected using ELISA kits (MultiScience, China) according to the manufacturer's instructions.

### 2.6. Lung Histopathology and Immunohistochemistry Analysis

The upper lobe of left lung without lavage was immediately fixed with 10% formaldehyde for 48 h, embedded in paraffin, sliced into 4 *μ*m thick sections, and stained with hematoxylin and eosin solution (H&E) or Alcian blue periodic acid Schiff (AB/PAS) for histopathologic examination and goblet cell metaplasia of bronchial epithelium.

For analyzing the effects of GFDHP on mucus secretion, the Mucin5ac (Muc5ac) protein expression levels in the airway epithelium were performed by immunohistochemistry (IHC) analysis [18]. Change in the MAPK pathway in the airway was investigated on the basis of detecting Muc5ac regulatory proteins including extracellular signal-regulated kinase (ERK)/p-ERK/specificity protein1(SP1) through IHC. After antigen was repaired and endogenous peroxidase was inactivated and blocked, the sections were incubated overnight at 4°C with anti-Muc5ac (Abcam, USA, dilution 1 : 200), anti-ERK (dilution 1 : 200), anti-p-ERK (dilution 1 : 500), and anti-SP1 (dilution 1 : 200) antibodies (Proteintech, China) successively. The sections were then incubated with horseradish peroxidase- (HRP-) conjugated second antibodies and developed with 3, 3-diaminobenzidine tetrahydrochloride. The degree of brown stains reflected the expression levels of the target proteins.

### 2.7. Real-Time Quantitative PCR Analysis

Total RNA was isolated from a part of the left lung with an animal total RNA isolation kit (Sangon, China) according to the manufacturer's instructions for the detection of Muc5ac mRNA expression level. The cDNA was synthesized using Hifair ii 1st strand cDNA synthesis kit (Yeasen, China), and real-time PCR reactions were performed using Hieff qPCR SYBR Green master mix (Yeasen, China) in CFX96 real-time system (Bio-Rad, USA). The specific primers of Muc5ac were as follows: Muc5ac-qF: GTGGTTTGACACTGACTTCCC; Muc5ac-qR: CTCCTCTCGGTGACAGAGTCT. The primers of GAPDH were as follows: GAPDH-qF: TGTGTCCGTCGTGGATCTGA; GAPDH-qR: TTGCTGTTGAAGTCGCAGGAG. The relative expression of Muc5ac was calculated using the 2^−ΔΔCT^ method.

### 2.8. Western Blot Analysis

Total proteins were extracted from part of the mouse left lung with radio immunoprecipitation assay (RIPA) lysis buffer (containing 1 mM PMSF). The protein levels of each sample were determined with a BCA assay kit. Each denatured protein sample (40 *μ*g) was separated with 10% sodium dodecyl sulfate polyacrylamide gel electrophoresis and electrotransferred to polyvinylidene fluoride membranes. The membranes were preblocked with 5% bovine serum albumin in tris buffered saline tween (TBST) for 1 h at room temperature and then incubated overnight at 4°C with anti-Muc5ac (56 kDa, Abcam, USA), anti-GAPDH (36 kDa), anti-ERK (42/44 kDa), anti-p-ERK (42/44 kDa), anti-SP1 (95 kDa), and anti-Tubulin (52 kDa) antibodies (Proteintech, China) successively. After being washed three times with TBST, the membranes were incubated with HRP-conjugated second antibodies for 1 h at room temperature. The band of each protein was detected using Western blotting reagents and autoradiographed with 3300 Mini ChemiScope Series (Clinx Science Instruments, Shanghai, China). Band intensities were quantified with 3300 Mini ChemiScope image analysis software. The phosphorylation and expression levels of proteins in the Dex group were not analyzed by WB.

### 2.9. Statistical Analysis

All data were shown as mean ± standard deviation (SD). Data were analyzed and figures were drawn with GraphPad Prism 8 (GraphPad Software, USA). Statistical differences among groups were determined by one-way analysis of variance and Tukey's test. A *P* value of <0.05 was considered statistically significant.

## 3. Results

### 3.1. GFDHP Improved Mouse Pulmonary Function Induced by LPS/CS

**Figure 2:**
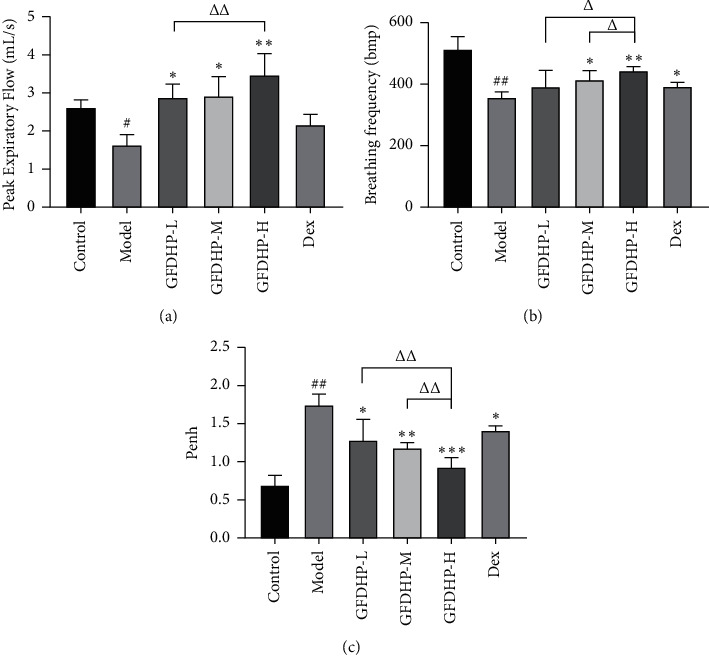
Effect of GFDHP on pulmonary function changes induced by LPS/CS in mice. (a) GFDHP improved peak expiratory flow, and (b) breathing frequency and alleviated (c) enhanced pause (PenH) examined by unrestrained whole-body plethysmography. The values were indicated as mean ± SD (*n* = 15). ^#^*P* < 0.05, ^###^*P* < 0.005, compared with the control group; ^∗^*P* < 0.05, ^∗∗^*P* < 0.01, ^∗∗∗^*P* < 0.005 compared with the model group; ^Δ^*P* < 0.05, ^ΔΔ^*P* < 0.01, compared with the GFDHP-H group.

To assess effects of GFDHP on mouse pulmonary function, a noninvasive pneumotachograph was used in the mice of each group. The respiratory function parameters PEF and *f* decreased significantly compared with the control group, but the bronchial reactivity parameter PenH increased significantly in the model group (*P* < 0.05). These results suggested LPS/CS-induced damage of the pulmonary function. Compared with the model group, PEF, *f*, and PenH were restored in the GFDHP-treated groups in a dose-dependent manner compared with those in the model group (*P* < 0.05) (Figure 2). The results showed that GFDHP improved pulmonary function deterioration induced by LPS/CS.

### 3.2. GFDHP Alleviated Pulmonary Hyperpermeability and Edema Induced by LPS/CS

**Figure 3:**
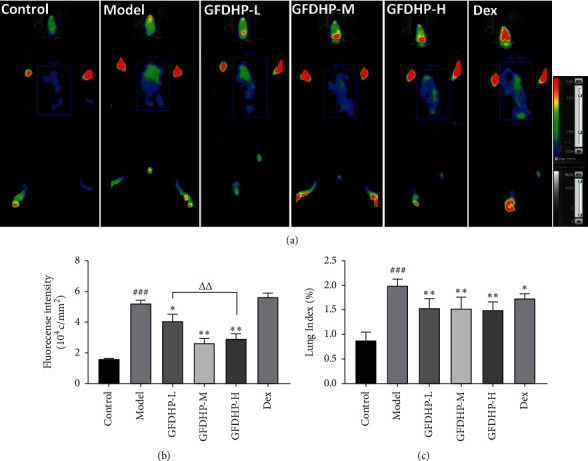
Effect of GFDHP on pulmonary hyperpermeability and edema induced by LPS/CS in mice. (a) GFDHP decreased the leakage of Evans blue into the lung according to *in vivo* imaging and (b) fluorescence intensities which were calculated with OPT inherent software (*n* = 5). (c) GFDHP reduced the lung indexes (*n* = 10). The values were indicated as mean ± SD. ^###^*P* < 0.005, compared with the control group; ^∗^*P* < 0.05, ^∗∗^*P* < 0.01 compared with the model group; ^ΔΔ^*P* < 0.01, compared with the GFDHP-H group.

*In vivo* Evans blue imaging can be used in evaluating damage and the hyperpermeability of mouse lung tissues. Fluorescence intensities in the lung tissues of the model mice markedly increased compared with the control group (*P* < 0.05) (Figure 3(a)). However, the leakage of the Evans blue in the lung tissues of the GFDHP-treated mice was significantly reduced compared with the model group. Similarly, LPS/CS increased lung indexes relative to those in the control group (*P* < 0.05) (Figure 3(b)), but GFDHP reduced the lung indexes significantly (*P* < 0.05). The results indicated that GFDHP inhibited pulmonary hyperpermeability and edema induced by LPS/CS.

### 3.3. GFDHP Reduced Mouse Pulmonary Histopathological Changes Induced by LPS/CS

**Figure 4:**
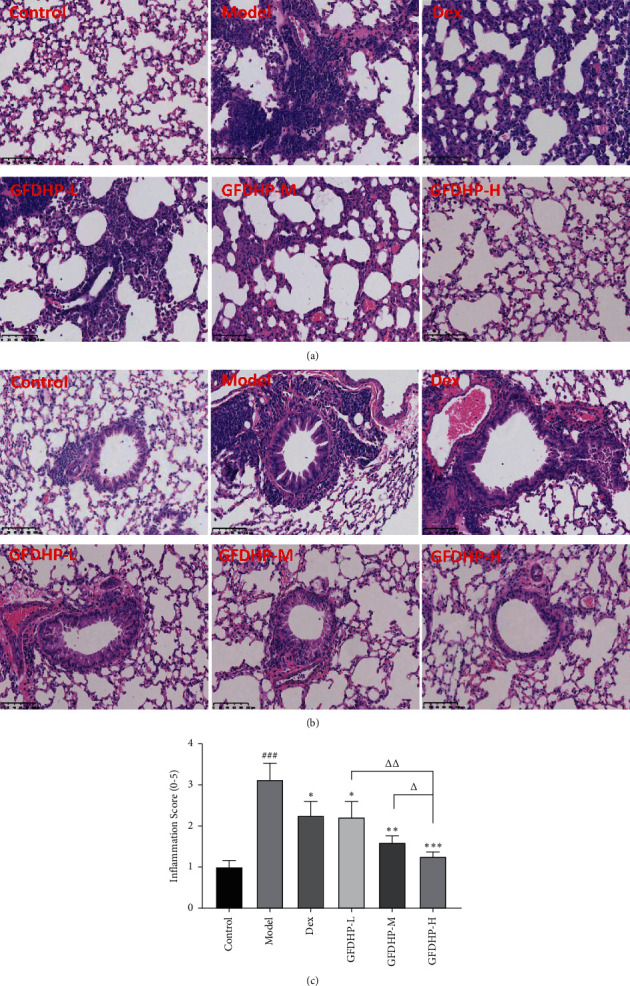
Effect of GFDHP on lung histological changes induced by LPS/CS in mice. (a) Pathological changes in the alveoli and (b) the airway structure analyzed by hematoxylin and eosin staining (H&E, magnification × 200), (c) the scoring of the extent of inflammation by the quantitative analysis of inflammatory cell infiltration in the lung parenchyma (*n* = 5). ^###^*P* < 0.005, compared with the control group; ^∗^*P* < 0.05, ^∗∗^*P* < 0.01, ^∗∗∗^*P* < 0.005 compared with the model group; ^Δ^*P* < 0.05, ^ΔΔ^*P* < 0.01, compared with the GFDHP-H group.

For the evaluation the effects of GFDHP on lung histology, the left lung sections from each group were subjected to H&E staining. In the lung tissues of the control group, the integrity of the structures of the pulmonary alveoli and capillary were maintained and showed no edema. The bronchus had appropriate wall thickness and did not have any infiltrated inflammatory cells. However, obvious histopathological changes were found in the model group. A mass of lymphocytes and neutrophils infiltrated the alveoli. Moreover, the COPD features, such as mucus hypersecretion, capillary congestion, thickened airway, hyperplastic airway epithelial cells, and lumen obstructed by mucus and cell debris, were observed in these lung tissues (Figure 4). Treatment with GFDHP especially in high dose improved the LPS/CS-induced airway obstruction and suppressed inflammatory cells infiltration, indicating its protective effects against LPS/CS-induced chronic lung injury. Inflammatory score confirmed that GFDHP alleviated pathological injury induced by LPS/CS, and the effect was in a dose-dependent manner (*P* < 0.05).

### 3.4. GFDHP Suppressed Mouse Inflammatory Cells and Cytokines Release in BALF Induced by LPS/CS

**Figure 5:**
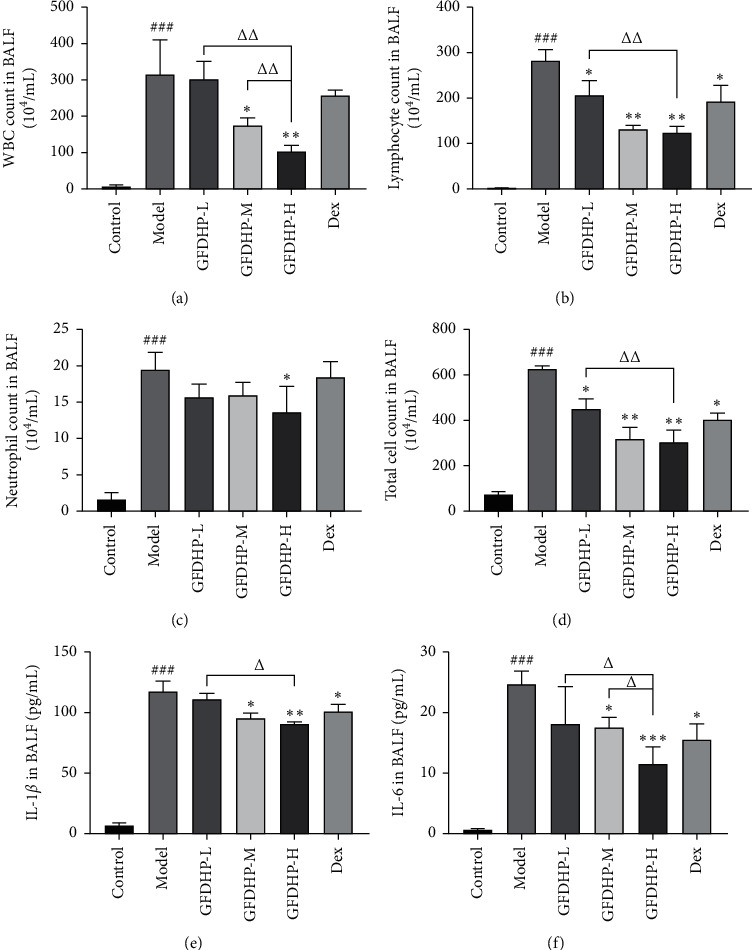
Effect of GFDHP on BALF inflammatory cells and cytokines induced by LPS/CS in mice. (a) Changes of white blood cell (WBC) count, (b) lymphocyte count, (c) neutrophil count, (d) total cell count, (e) IL-1*β*, and (f) IL-6 in BALF. The values were indicated as mean ± SD (*n* = 10). ^###^*P* < 0.005, compared with the control group; ^∗^*P* < 0.05, ^∗∗^*P* < 0.01, ^∗∗∗^*P* < 0.005 compared with the model group; ^Δ^*P* < 0.05, ^ΔΔ^*P* < 0.01, compared with the GFDHP-H group.

For the investigation of the effect of GFDHP on airway inflammation, the counts of inflammatory cells (including total cells, white blood cells (WBC), lymphocytes, and neutrophils) and the levels of cytokines (IL-1*β* and IL-6) in BALF were detected. As shown in Figure 5, the counts of total cells, WBCs, lymphocytes, and neutrophils and the levels of IL-1*β* and IL-6 were elevated significantly in the model group relative to those in the control group (*P* < 0.05). However, GFDHP significantly reduced the number of inflammatory cells and inhibited cytokine release in a dose-dependent manner (*P* < 0.05). These results were consistent with the findings of H&E analysis.

### 3.5. GFDHP Decreased Airway Goblet Cell Hyperplasia Induced by LPS/CS

**Figure 6:**
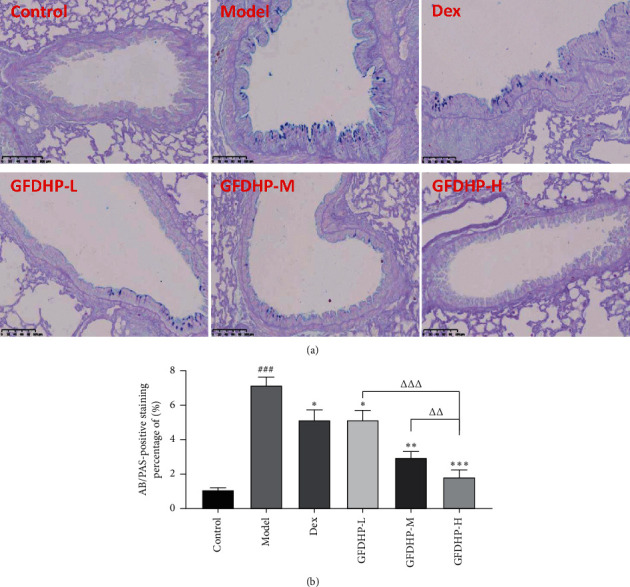
Effect of GFDHP on bronchial epithelium goblet cell metaplasia induced by LPS/CS in mice. (a) AB/PAS staining (magnification × 200), (b) quantification of goblet cell hyperplasia (*n* = 10). The values were indicated as mean ± SD (*n* = 10). ^###^*P* < 0.005, compared with the control group; ^∗∗^*P* < 0.01, ^∗∗∗^*P* < 0.005 compared with the model group; ^ΔΔ^*P* < 0.01, ^ΔΔΔ^*P* < 0.005, compared with the GFDHP-H group.

After staining with the AB/PAS dye, the mucus secreted by the activated goblet cells appeared blue. This result can be used in assessing the activation of goblet cells (i.e., cell hyperplasia). As shown in Figure 6, the positive cells were hardly found in the control group, indicating the inactivated goblet cells that did not secrete mucus. The positive staining areas significantly increased in the airway epithelium of the model group compared with those in the control group but were significantly decreased in the GFDHP-treated group in a dose-dependent manner (*P* < 0.05). This result demonstrated that the hyperplasia of goblet cells was induced by long-term exposure of LPS/CS but suppressed by GFDHP.

### 3.6. GFDHP Inhibited Airway Mucus Hypersecretion Induced by LPS/CS

**Figure 7:**
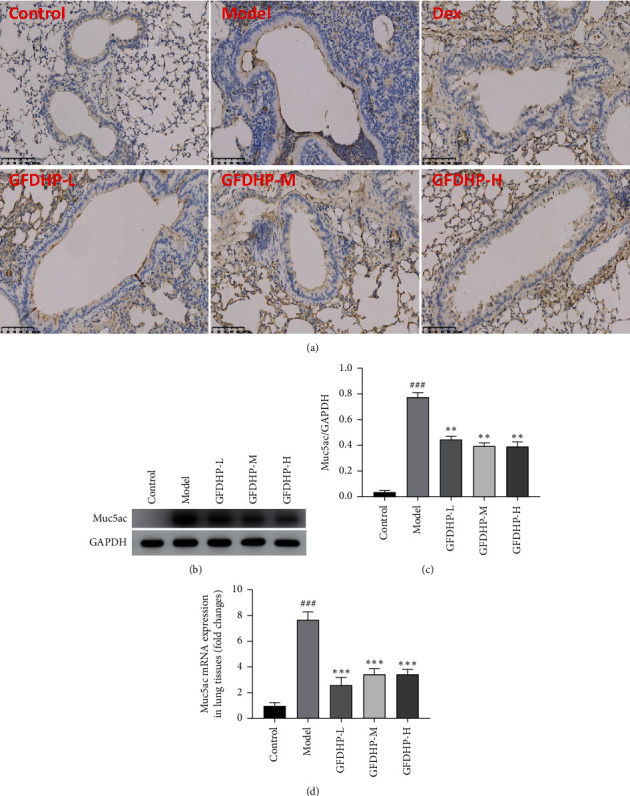
Effect of GFDHP on the secretion and expression of Muc5ac in the lung. (a) Immunohistochemical staining of Muc5ac in the airway (magnification × 200). The protein and mRNA expression levels of Muc5ac in the lung tissue were detected with (b) Western blot and (d) real-time quantitative PCR; (c) the proportions of Muc5ac to GAPDH optical density were analyzed with a software. The values were indicated as mean ± SD (*n* = 10). ^###^*P* < 0.005, compared with the control group; ^∗∗^*P* < 0.01, ^∗∗∗^*P* < 0.005 compared with the model group.

The hypersecretion of mucus is one of the COPD features in patients with COPD. Thus, to investigate the therapeutic effects of GFDHP in COPD mice, secretion and expression of Muc5ac in lung tissue were determined by IHC, qPCR, and WB analysis. In the airway of the control group, brown stains were hardly detected, while the levels of brown stains in the model airway epithelium were much higher than that in the control group (*P* < 0.05). Stain depths decreased with increasing GFDHP dosages compared with those in the model group (Figure 7(a)). Furthermore, qPCR and Western blot (WB) data (Figures 7(b)–7(d)) show that the expression levels of Muc5ac mRNA and protein in the lung tissues of the model mice were significantly higher than those in the control group (*P* < 0.05), but they were significantly reduced after GFDHP treatment (*P* < 0.05). These results indicated that GFDHP inhibited Muc5ac expression and secretion induced by LPS/CS in mice.

### 3.7. GFDHP Downregulated the Phosphorylation of ERK and Expression of SP1 to Inhibit Muc5ac

**Figure 8:**
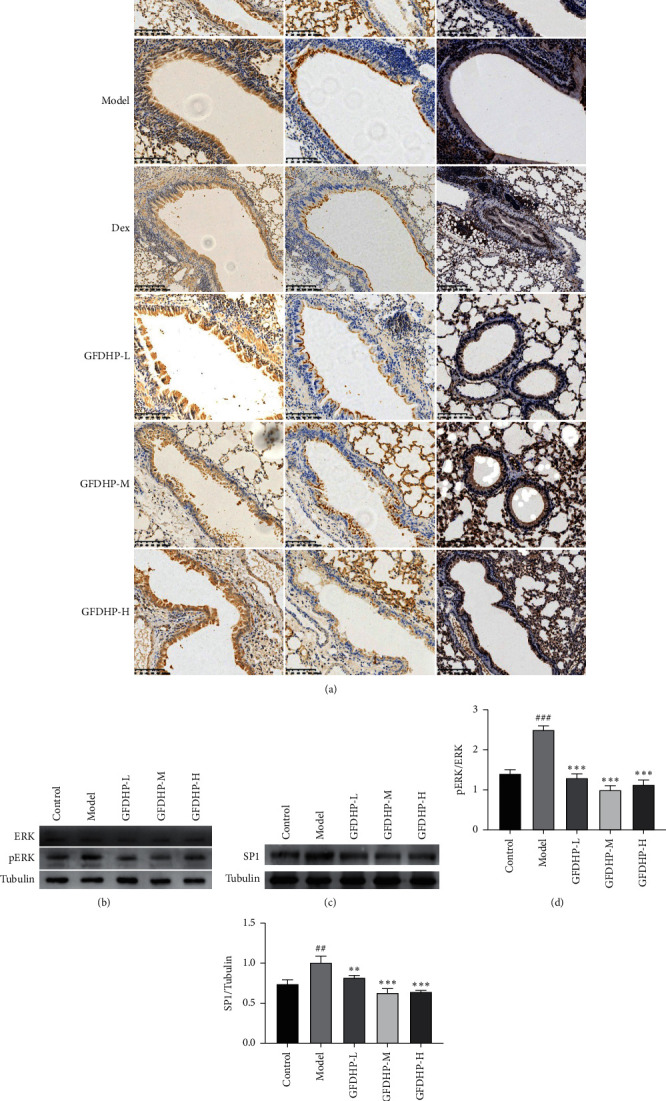
Effect of GFDHP on phosphorylation of ERK and expression of SP1 in lung. (a) Immunohistochemical staining of ERK, p-ERK, and SP1 in the airway (magnification × 200). (b) The phosphorylation of ERK and (c) protein level of SP1 was detected with Western blot and (d-e) quantification. The values were indicated as mean ± SD (*n* = 3). ^##^*P* < 0.01, ^###^*P* < 0.005, compared with the control group; ^∗∗^*P* < 0.01, ^∗∗∗^*P* < 0.005 compared with the model group.

As shown in Figure 8, p-ERK level in the model group significantly increased compared with that in the control group, but there was no difference in p-AKT level between the control and model groups (data not shown). However, GFDHP treatment significantly downregulated p-ERK in the lung tissues of the COPD mice in a dose-dependent manner (*P* < 0.05). Then, as presented by IHC and WB analysis, the expression of SP1, a key regulator of CS-induced Muc5ac mRNA transcription, was also reduced by GFDHP (*P* < 0.05). This result demonstrated that GFDHP inhibited LPS/CS-induced lung inflammation in COPD mice by inactivating the ERK-SP1 pathway.

## 4. Discussion

**Figure 9:**
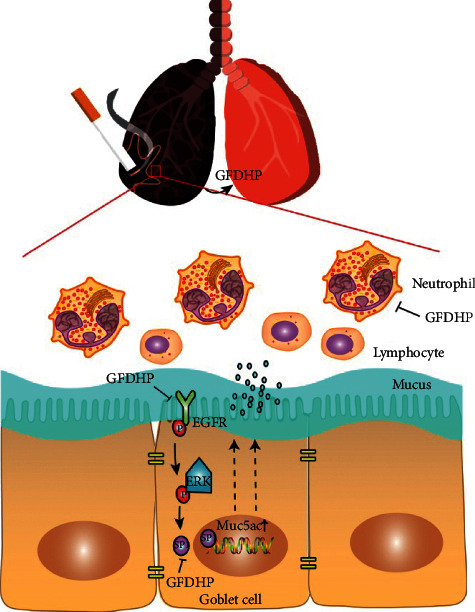
Summary diagram showing the protective effects of GFDHP against LPS/CS-induced airway inflammation and mucus hypersecretion through the suppression of Muc5ac expression and inactivation of the ERK-SP1 pathway.

GFDHP is a classical Chinese herbal formula dispensed in the Synopsis of Golden Chamber written by Zhang Zhongjing approximately 2000 years ago and was called the Shenqi pill. According to the theory of TCM, GFDHP improved lung immunity and renal hypofunction and consequently prevented chronic pneumonia and bronchitis, as well as nocturia [16] and spermatorrhea [19]. GFDHP exhibited great therapeutic effects in patients with chronic lung diseases, especially those with COPD at the stable stage [20], significantly shortening the treatment course, improving the quality of life, and reducing complications. However the mechanism of GFDHP has not been extensively explored. In this study, a COPD mouse model was established by LPS intranasal inhalation in combination with continuous CS exposure for 6 weeks for the impairment of pulmonary function. Treatment with GFDHP at doses of 0.4–1.6 g/kg alleviated declined peak expiratory flow, enhanced breathing frequency, and increased pause of COPD mice. Moreover, GFDHP effectively alleviated pulmonary edema induced by CS/LPS by inhibiting pulmonary hyperpermeability and mucus hypersecretion. The results of WB and IHC analysis showed that GFDHP significantly inhibited the expression levels of Muc5ac mRNA and protein and downregulated the activation of the ERK-SP1 pathway (Figure 9).

Airway inflammation is a consistent feature of COPD and plays an important role in the pathogenesis and progression of COPD [21]. Sustained CS exposure induced the accumulation of harmful substances and particles in the lung tissues of patients with COPD, thereby recruiting immune cells (such as neutrophils, lymphocytes, and leukocytes) to remove the stimulus but causing the release of cytokines and inflammatory mediators, which caused the continued inflammatory reaction and abnormal immune responses and mucous overproduction and remodeling of the airway and vasculature [2]. Therefore, anti-inflammatory therapy can be one of the potential strategies for treatment of COPD. In the present study, GFDHP effectively relieved airway inflammation induced by LPS/CS as it significantly decreased neutrophils, lymphocytes, and leukocytes counts and suppressed the levels of IL-1*β* and IL-6 released in BALF. Although inflammatory cytokines (including IL-1*β* and IL-6) eliminate foreign particles and irritants, their continued release causes tissue injury and increases pulmonary permeability [22, 23]. Indeed, after long-term CS exposure, the exudative amount of Evens blue considerably increased in the lung tissues of the model mice, indicating increased pulmonary microvascular permeability, whereas treatment with GFDHP significantly decreased the exudation of dying materials, reduced pulmonary edema, and then improved lung ventilation function.

Respiratory mucus layers, which serve as the lining on the surface of the airway epithelium, trap and remove inhaled insults from airways though mucociliary movement [24]. Normal mucus is a liquid solution composed of 97% water and 3% contents (including mucins, other proteins, lipids, salts, and debris) [25]. In patients with COPD, the overexpression and hypersecretion of mucins can increase the concentrations of the contents up to 15%, resulting in viscous and elastic mucus being cleared difficultly and adhering more readily to the airway [26]. Therefore, excess mucus aggravates airway obstruction, increases airway reactivity, and reduces pulmonary function [27]. Approximately 95% of the total mucins secreted in the airway epithelium was produced by goblet cells and can be detected with AB/PAS staining [28]. We found high positive expressions presented in the lung tissues of the COPD mice, but it was hardly detected in the normal mice, indicating the hyperplasia of goblet cells and mucus hypersecretion in the COPD mice. However, treatment with GFDHP significantly decreased positive expressions induced by AB/PAS staining, indicating inhibition effect of GFDHP on airway goblet cell hyperplasia. Meanwhile GFDHP significantly inhibited the mRNA and protein expression levels of Muc5ac, which were significantly upregulated in lung tissues of the COPD mice. These results demonstrated that GFDHP inhibited the transcription and synthesis of Muc5ac and subsequently reduced mucus overproduction.

Many signaling pathways are involved in abnormal Muc5ac production [29]. The epidermal growth factor receptor can be activated by LPS or CS or both, which in turn initiates the Ras/MEK/ERK signaling of mitogen-activated protein kinase (MAPK) cascade [30]. Actually, the MAPK signaling pathway is positively correlated with the progressive self-amplifying disordering of airway epithelial differentiation and function during the pathogenesis of COPD, including the overproduction of Muc5ac [31]. Indeed, phosphorylation ERK is a critical factor in the activation of SP1 in nucleus [32], and SP1 can bind to the promoter region of Muc5ac to induce Muc5ac expression [33]. Therefore, ERK-SP1 is a definite pathway that activates Muc5ac expression. In this study, the expression levels of Muc5ac, p-ERK, and SP1 were significantly increased in the lung tissues of COPD mice. The result was associated with previous reports [34, 35]. However, treatment with GFDHP significantly downregulated the expression levels of those proteins, indicating that GFDHP reduced mucus overproduction by inhibiting the ERK-SP1-Muc5ac pathway.

The effect of GFDHP on COPD mice induced by LPS/CS in this study verified its clinical efficacy and revealed underling mechanism. Nevertheless, some worth noticing points still need to be explored. Firstly, corticosteroids including dexamethasone have been used in the treatment of COPD for ≥40 years with moderate overall benefit in clinical and almost all models of smoking or LPS-induced airway inflammation [36]. However, there are a variety of dosages from 0.25 to 2 mg/kg and administrated periods from 3 to 90 days in COPD animal models as previously reported [18, 37–39]. Particularly, dexamethasone had obvious side effects during long-term treatment, such as weight loss [36] and locomotor activity decreased in animal models, which could reverse the benefits of dexa treatment. The effect of dexamethasone was less than GFDHP in some test indicators in this study, such as pulmonary function [38] and mucin secretion. The dosage and administrated period should be considered for further study. Secondly, GFDHP is made of eight raw medicinal materials, and its components are complex. It is necessary to further study that which one or several components play the major role. In addition, other signaling pathways might be involved in antimucus effect of GFDHP besides ERK-SP1-Muc5ac. Anyway, GFDHP is still a classic prescription worthy of further investigation for COPD therapy.

## 5. Conclusion

In summary, GFDHP reduced mucus overproduction and attenuated pulmonary ventilation function in LPS/CS-induced COPD mice by suppressing Muc5ac and the ERK-SP1 signaling pathway. Therefore, GFDHP can be used in COPD treatment.

## Data Availability

The data used to support the findings of this study are available in the article and Supplementary Materials.

## Abbreviations

AB/PAS: Alcian blue periodic acid Schiff
BALF: Bronchoalveolar lavage fluid
COPD: Chronic obstructive pulmonary disease
ERK: Extracellular signal-regulated kinase
H&E: Hematoxylin and eosin solution
IHC: Immunohistochemistry
IL: Interleukin
GFDHP: Guifu Dihuang pill
KDY: Kidney yang deficiency
LPS/CS: Lipopolysaccharide/cigarette smoking
MAPK: Mitogen-activated protein kinase
Muc5ac: Mucin5ac
PEF: Peak expiratory flow
PenH: Enhanced pause
SP1: Specificity protein1
TCM: Traditional Chinese medicine
TNF-*α*: Tumor necrosis factor-*α*
WB: Western blot
WBC: White blood cells.

## Supplementary Materials

Supplementary Materials Supplementary data 1: fingerprints of Guifu Dihuang pills (GFDHP) detected using UPLC-Q/TOF-MS (Figure S1). Supplementary data 2: name and retention time (RT) of standards (Table S1). Supplementary data 3: components of Guifu Dihuang pills (Table S2).
