# Supplementary material for: Guifu Dihuang Pills Ameliorated Mucus Hypersecretion by Suppressing Muc5ac Expression and Inactivating the ERK-SP1 Pathway in Lipopolysaccharide/Cigarette Smoke-Induced Mice
Source: Evid Based Complement Alternat Med. 2021 Nov 3;2021:9539218. doi: 10.1155/2021/9539218 (PMC8580658; doi:10.1155/2021/9539218)
Supplement: Supplementary Materials — Supplementary data 1: fingerprints of Guifu Dihuang pills (GFDHP) detected using UPLC-Q/TOF-MS (Figure S1). Supplementary data 2: name and retention time (RT) of standards (Table S1). Supplementary data 3: components of Guifu Dihuang pills (Table S2). [file 9539218.f1.docx]

A


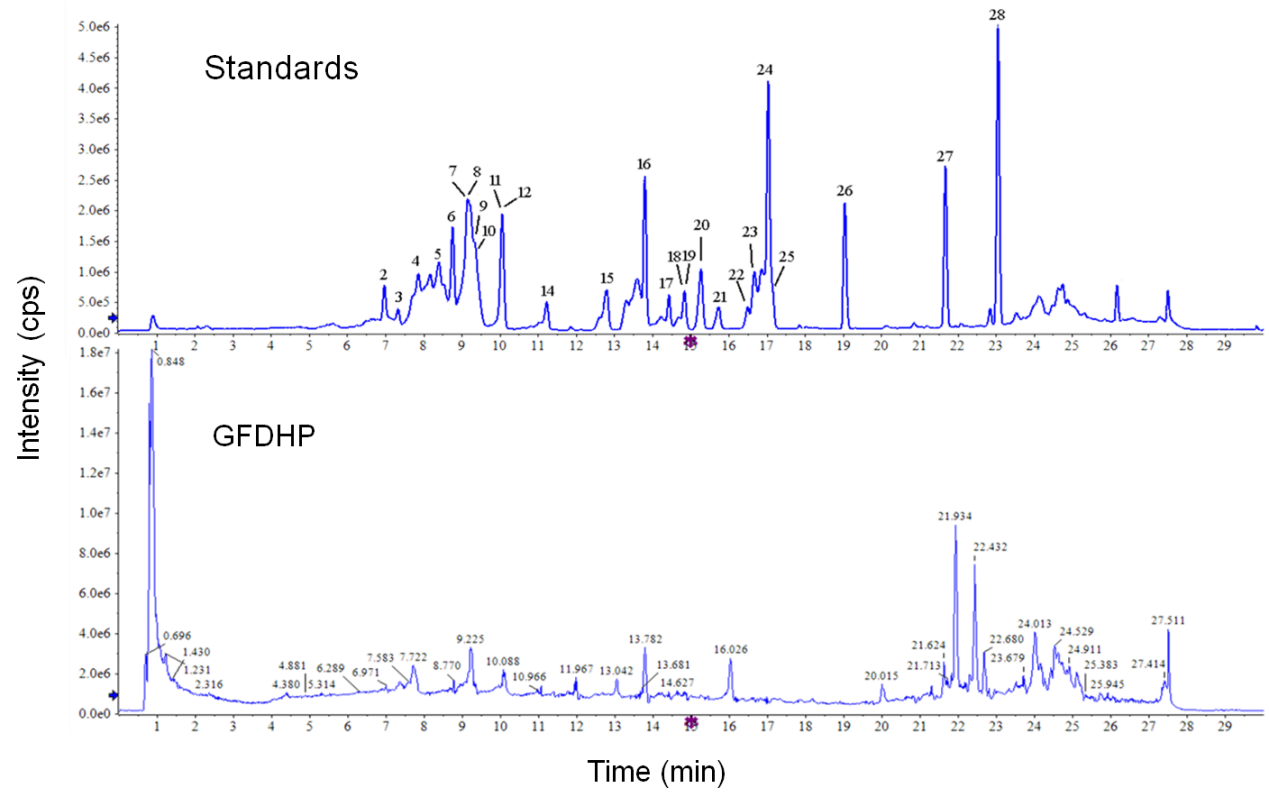


B


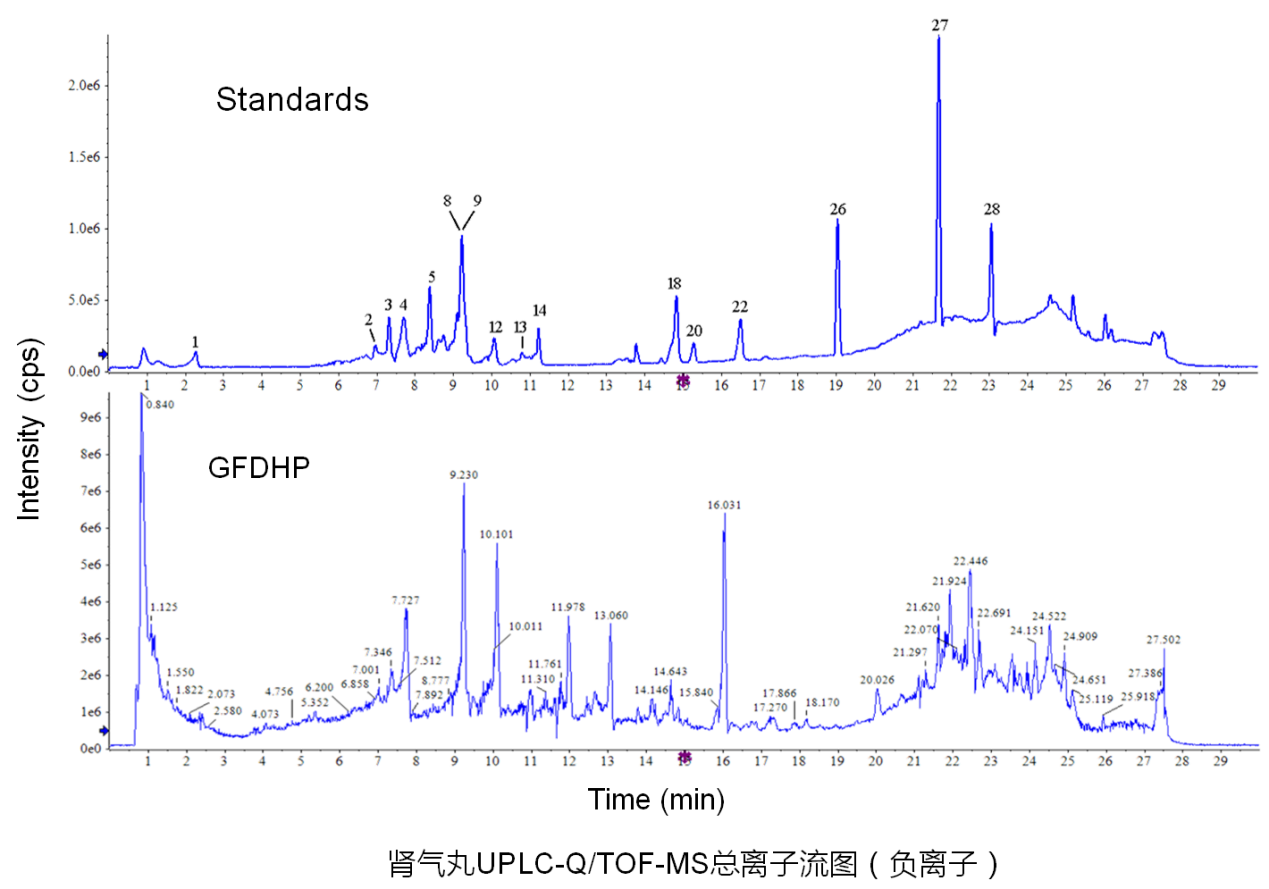


Fig.S1 Fingerprints of Guifu Dihuang pills (GFDHP) detected using UPLC-Q/TOF-MS. (A) the results of positive ion mass spectrum and (B) negative ion mass spectrum.

Table.S1 Name and retention time(RT) of Standards.

| No. | Name | RT(min) | No. | Name | RT(min) |
| --- | --- | --- | --- | --- | --- |
| 1 | Gallic acid | 2.28 | 15 | Coumarin | 12.79 |
| 2 | Noraconitine | 6.97 | 16 | Benzoylmesaconine | 13.76 |
| 3 | Loganic acid | 7.3 | 17 | Benzoylaconitine | 14.44 |
| 4 | Morroniside | 7.65 | 18 | Quercetin | 14.81 |
| 5 | Swertiamarin | 8.35 | 19 | Benzoylhypacoitine | 14.88 |
| 6 | Fuziline | 8.75 | 20 | Cinnamic acid | 15.27 |
| 7 | Neoline | 9.13 | 21 | Cinnamaldehyde | 15.72 |
| 8 | Loganin | 9.19 | 22 | Isorhamnetin | 16.47 |
| 9 | Sweroside | 9.29 | 23 | Paeonol | 16.64 |
| 10 | Vanillin | 9.43 | 24 | Aconitine | 17.02 |
| 11 | Talatizamine | 10.02 | 25 | O-methoxycinnamaldehyde | 17.13 |
| 12 | Paeoniflorin | 10.06 | 26 | Saikosaponin A | 18.97 |
| 13 | Ellagic Acid | 10.78 | 27 | Alisol A | 21.66 |
| 14 | Hyperin | 11.21 | 28 | Alisol B | 23.05 |

Table.S2 Components of Guifu Dihuang pills

| Component Name | Adduct | Area | RT  (min) | Formula | Precursor Mass | Found At Mass | Mass Error (ppm) |
| --- | --- | --- | --- | --- | --- | --- | --- |
| Gallic acid | [M-H]- | 4354000 | 2.39 | C7H6O5 | 169.014 | 169.0144 | 1 |
| Loganic acid | [M-H]- | 3125000 | 7.36 | C16H24O10 | 375.13 | 375.1296 | -0.3 |
| Morroniside | [M-H]- | 3439000 | 7.72 | C17H26O11 | 405.14 | 405.14 | -0.6 |
| Fuziline | [M+H]+ | 2584000 | 8.77 | C24H39NO7 | 454.28 | 454.28 | 0.2 |
| Neoline | [M+H]+ | 585000 | 9.16 | C24H39NO6 | 438.285 | 438.2852 | 0.5 |
| Loganin | [M+HCOO]- | 20960000 | 9.23 | C17H26O10 | 435.151 | 435.1506 | -0.4 |
| Sweroside | [M+HCOO]- | 2588000 | 9.35 | C16H22O9 | 403.125 | 403.1247 | 0.4 |
| Vanillin | [M+H]+ | 13350 | 9.49 | C8H8O3 | 153.055 | 153.0549 | 2 |
| Talatizamine | [M+H]+ | 136400 | 10.06 | C24H39NO5 | 422.29 | 422.2905 | 0.8 |
| Paeoniflorin | [M+HCOO]- | 8871000 | 10.1 | C23H28O11 | 525.161 | 525.1612 | -0.3 |
| Ellagic Acid | [M-H]- | 1688000 | 10.82 | C14H6O8 | 300.999 | 300.9988 | -0.6 |
| Hyperin | [M+H]+ | 6665 | 11.34 | C21H20O12 | 465.103 | 465.1031 | 0.8 |
| Coumarin | [M+H]+ | 223800 | 12.8 | C9H6O2 | 147.044 | 147.0439 | -0.8 |
| Benzoylmesaconine | [M+H]+ | 10390000 | 13.78 | C31H43NO10 | 590.296 | 590.2959 | -0.1 |
| Benzoylaconitine | [M+H]+ | 1819000 | 14.42 | C32H45NO10 | 604.312 | 604.3118 | 0.4 |
| Quercetin | [M-H]- | 188000 | 14.82 | C15H10O7 | 301.035 | 301.0348 | -2 |
| Benzoylhypacoitine | [M+H]+ | 2363000 | 14.84 | C31H43NO9 | 574.301 | 574.301 | -0.1 |
| Cinnamic acid | [M+H]+ | 28430 | 15.26 | C9H8O2 | 149.06 | 149.0597 | 0.1 |
| Cinnamaldehyde | [M+H]+ | 7394 | 15.72 | C9H8O | 133.065 | 133.0649 | 0.8 |
| Isorhamnetin | [M-H]- | 38730 | 16.47 | C16H12O7 | 315.051 | 315.0505 | -1.6 |
| Paeonol | [M+H]+ | 703400 | 16.64 | C9H10O3 | 167.07 | 167.0702 | -0.3 |
| Aconitine | [M+H]+ | 93860 | 17.03 | C34H47NO11 | 646.322 | 646.3227 | 0.8 |
| O-methoxycinnamaldehyde | [M+H]+ | 70350 | 17.11 | C10H10O2 | 163.075 | 163.0755 | 0.7 |
| Saikosaponin A | [M+HCOO]- | 43190 | 18.98 | C42H68O13 | 825.464 | 825.463 | -1.5 |
| Alisol A | [M+HCOO]- | 3962000 | 21.63 | C30H50O5 | 535.364 | 535.3644 | 0.8 |
| Alisol B | [M+HCOO]- | 395300 | 22.84 | C30H48O4 | 517.353 | 517.3538 | 0.7 |
